# Supplementary material for: Assessment of air pollution and air quality perception mismatch using mobility-based real-time exposure
Source: PLoS One. 2024 Feb 27;19(2):e0294605. doi: 10.1371/journal.pone.0294605 (PMC10898763; doi:10.1371/journal.pone.0294605)
Supplement: S1 Table — (DOCX) [file pone.0294605.s001.docx]

**S1 Table. Descriptive statistics of participants’ characteristics.**

| **Personal attributes** | **Code** | **Percent** | **SSP** | **TSW** |
| --- | --- | --- | --- | --- |
| **House ownership** |  |  |  |  |
| Rent | 0 | 59.0% | 62.9% | 55.2% |
| Own without mortgage | 1 | 21.0% | 20.0% | 21.9% |
| Own with mortgage | 1 | 19.0% | 15.2% | 22.9% |
| **Living space (ft2)** |  |  |  |  |
| <100 | 1 | 3.3% | 6.7% |  |
| 100-300 | 2 | 28.1% | 37.1% | 19.0% |
| 301-500 | 3 | 44.3% | 45.7% | 42.9% |
| 501-800 | 4 | 21.4% | 9.5% | 33.3% |
| 801-1100 | 5 | 1.4% | 1.0% | 1.9% |
| >1100 | 6 | 1.4% |  | 2.9% |
| **Family member** |  |  |  |  |
| 1-2 | - | 29.5% | 42.9% | 16.2% |
| 3-4 | - | 59.0% | 50.5% | 67.6% |
| 5-6 | - | 11.4% | 6.7% | 16.2% |
| **Respiratory symptoms** |  |  |  |  |
| 0-6 | - | 46.7% | 38.1% | 55.2% |
| 7-12 | - | 48.6% | 59.0% | 38.1% |
| 13-18 | - | 2.9% | 0.0% | 5.7% |
| **Physical exercise days in one week (Physical exercise)** |  |  |  |  |
| ≤3 | - | 82.9% | 87.6% | 78.1% |
| 4-7 | - | 17.1% | 12.4% | 21.9% |
