# Supplementary material for: Assessment of air pollution and air quality perception mismatch using mobility-based real-time exposure
Source: PLoS One. 2024 Feb 27;19(2):e0294605. doi: 10.1371/journal.pone.0294605 (PMC10898763; doi:10.1371/journal.pone.0294605)
Supplement: S2 Table — (DOCX) [file pone.0294605.s002.docx]

S2 Table. Regression results of association between influencing factors and perception of air pollution using residence-based methods.

|  | RB: 50m | RB: 50m-100m | RB: 100m-150m | RB: 150m-200m | RB: 200m-250m | RB: 250m-300m | RB: 300m-350m | RB: 350m-400m | RB: 400m-450m | RB: 450m-500m |
| --- | --- | --- | --- | --- | --- | --- | --- | --- | --- | --- |
| Variables | Coef. | Coef. | Coef. | Coef. | Coef. | Coef. | Coef. | Coef. | Coef. | Coef. |
| (Intercept) | 3.437 | 3.896. | 3.397* | 4.524** | 4.670** | 3.894* | 3.829* | 4.406** | 4.612** | 4.469** |
| PM2.5 | 0.004 | 0.005 | -0.005 | -0.008 | -0.001 | -0.001 | -0.002 | 0.002 | 0.001 | 0.001 |
| Gender(ref.:Male) |  |  |  |  |  |  |  |  |  |  |
| Female | -0.376 | -0.402 | -0.290* | -0.276* | -0.280* | -0.273* | -0.269. | -0.262. | -0.265. | -0.266. |
| Neighborhood(ref.:SSP) | | |  |  |  |  |  |  |  |  |
| TSW | -0.434 | -0.419 | -0.259 | -0.328. | -0.097 | -0.176 | 0.008 | -0.185 | -0.131 | -0.069 |
| Age | 0.000 | 0.000 | -0.013 | -0.018* | -0.022* | -0.022* | -0.023** | -0.023** | -0.023** | -0.023** |
| Education level | 0.044 | 0.047 | 0.053 | 0.033 | 0.031 | 0.043 | 0.032 | 0.030 | 0.046 | 0.046 |
| Marital status(ref.:Never married) | | |  |  |  |  |  |  |  |  |
| Divorced | 0.370 | 0.414 | 0.115 | 0.233 | 0.288 | 0.294 | 0.301 | 0.288 | 0.347 | 0.323 |
| Married | -0.012 | -0.015 | 0.222 | 0.280 | 0.347. | 0.335. | 0.364. | 0.321 | 0.337. | 0.371. |
| Widowed | -0.013 | 0.099 | 0.703 | 0.802. | 0.978* | 0.880. | 0.900. | 0.810 | 0.824. | 0.814. |
| Household income | 0.086 | 0.057 | 0.044 | 0.037 | 0.028 | 0.013 | 0.023 | 0.023 | 0.020 | 0.018 |
| Employment status(ref.:Employed) | | |  |  |  |  |  |  |  |  |
| Student | 0.468 | 0.535 | -0.160 | -0.294 | -0.310 | -0.437* | -0.400. | -0.438* | -0.410. | -0.364. |
| Unemployed | 0.150 | 0.124 | 0.000 | -0.016 | 0.003 | -0.042 | -0.054 | -0.019 | 0.018 | 0.004 |
| Family member | 0.030 | 0.041 | 0.021 | -0.007 | -0.016 | -0.020 | -0.012 | -0.023 | -0.029 | -0.028 |
| House Ownership(ref.: rent) | | |  |  |  |  |  |  |  |  |
| Own without mortgage | -0.254 | -0.355 | 0.061 | -0.016 | -0.002 | -0.079 | -0.098 | -0.108 | -0.126 | -0.118 |
| Own with mortgage | 0.297 | 0.233 | 0.058 | -0.012 | -0.120 | -0.120 | -0.161 | -0.196 | -0.208 | -0.201 |
| Living space | -0.065 | -0.022 | 0.013 | 0.049 | 0.057 | 0.083 | 0.083 | 0.099 | 0.106 | 0.111 |
| Physical exercise | 0.042 | 0.044 | 0.037 | 0.025 | 0.024 | 0.034 | 0.031 | 0.031 | 0.033 | 0.037 |
| Relative humidity | -0.024 | -0.027* | -0.006 | -0.011 | -0.013* | -0.013. | -0.013* | -0.015* | -0.015* | -0.014* |
| Temperature | 0.009 | 0.004 | -0.008 | -0.007 | -0.010 | -0.005 | -0.005 | -0.006 | -0.006 | -0.006 |
| Population density | 0.000 | 0.000 | 0.000 | 0.000 | 0.000 | 0.000 | 0.000 | 0.000 | 0.000 | 0.000 |
| Transportation land-use density | 28.680 | 31.900 | 89.600 | 39.940 | 9.332 | 103.700 | 80.900 | 67.150 | -1.726 | -43.170 |
| Green space density | -479.500 | 273.200 | 351.500* | 188.900 | 171.700 | 135.100 | 90.860 | -31.790 | -47.010 | -37.320 |
| Open space density | 3.424 | 4.311 | -3.824 | -3.337 | -1.311 | 1.502 | 2.093 | -1.011 | -3.014 | -2.208 |
| Facilities density | 596.700 | 956.900 | 1006.000. | -404.300 | 1009.000 | 511.700 | 2433.000* | 884.100 | 1269.000 | 2031.000. |
| Respiratory symptoms | 0.010 | -0.005 | 0.038 | 0.003 | 0.012 | 0.019 | 0.017 | 0.025 | 0.022 | 0.023 |
| Mental disorder | 0.042. | 0.043. | 0.059*** | 0.061*** | 0.063*** | 0.057*** | 0.059*** | 0.055*** | 0.055*** | 0.055*** |
| R^2^ | 0.336 | 0.350 | 0.298 | 0.224 | 0.230 | 0.217 | 0.236 | 0.215 | 0.220 | 0.223 |
| Adjusted R^2^ | 0.039 | 0.059 | 0.163 | 0.103 | 0.116 | 0.102 | 0.124 | 0.100 | 0.106 | 0.109 |
| Model’s P-value | 0.341 | 0.275 | 0.002 | 0.011 | 0.004 | 0.009 | 0.002 | 0.010 | 0.007 | 0.006 |

Signif. codes: ‘*******’: 0.001; ‘******’: 0.01; ‘*****’: 0.05; ‘.’: 0.1; ‘’: 1
