# Supplementary material for: Assessment of air pollution and air quality perception mismatch using mobility-based real-time exposure
Source: PLoS One. 2024 Feb 27;19(2):e0294605. doi: 10.1371/journal.pone.0294605 (PMC10898763; doi:10.1371/journal.pone.0294605)
Supplement: S3 Table — (DOCX) [file pone.0294605.s003.docx]

Supplementary Table 3. Regression results of association between influencing factors and perception of air pollution using mobility-based methods.

|  | MB: 50m | MB: 50m-100m | MB: 100m-150m | MB: 150m-200m | MB: 200m-250m | MB: 250m-300m | MB: 300m-350m | MB: 350m-400m | MB: 400m-450m | MB: 450m-500m |
| --- | --- | --- | --- | --- | --- | --- | --- | --- | --- | --- |
| Variables | Coef. | Coef. | Coef. | Coef. | Coef. | Coef. | Coef. | Coef. | Coef. | Coef. |
| (Intercept) | 4.297** | 4.116** | 4.010* | 3.926* | 3.722* | 3.220* | 3.268* | 3.392* | 3.847* | 4.130** |
| PM2.5 | 0.002 | 0.002 | 0.001 | 0.001 | 0.001 | 0.001 | 0.002 | 0.002 | 0.004 | 0.003 |
| Gender(ref.:Male) |  |  |  |  |  |  |  |  |  |  |
| Female | -0.237. | -0.227 | -0.234. | -0.243. | -0.262. | -0.239. | -0.245. | -0.235. | -0.233. | -0.257. |
| Neighborhood(ref.:SSP) | | |  |  |  |  |  |  |  |  |
| TSW | -0.217 | -0.162 | -0.197 | -0.161 | -0.120 | -0.059 | -0.084 | -0.081 | -0.045 | -0.077 |
| Age | -0.023** | -0.022* | -0.022* | -0.022* | -0.022* | -0.022** | -0.022** | -0.022* | -0.023** | -0.023** |
| Education level | 0.022 | 0.021 | 0.024 | 0.038 | 0.042 | 0.036 | 0.028 | 0.037 | 0.033 | 0.023 |
| Marital status(ref.:Never married) | | |  |  |  |  |  |  |  |  |
| Divorced | 0.304 | 0.315 | 0.225 | 0.218 | 0.284 | 0.315 | 0.272 | 0.235 | 0.296 | 0.309 |
| Married | 0.353. | 0.355. | 0.288 | 0.295 | 0.335. | 0.332. | 0.304 | 0.302 | 0.297 | 0.303 |
| Widowed | 0.854. | 0.829. | 0.722 | 0.839. | 0.862. | 0.917. | 0.865. | 0.842. | 0.820. | 0.825. |
| Household income | 0.030 | 0.031 | 0.031 | 0.016 | 0.019 | 0.020 | 0.015 | 0.012 | 0.020 | 0.022 |
| Employment status(ref.:Employed) | | |  |  |  |  |  |  |  |  |
| Student | -0.416. | -0.337 | -0.412. | -0.448* | -0.459* | -0.403. | -0.437* | -0.432* | -0.425. | -0.389. |
| Unemployed | -0.030 | -0.019 | -0.036 | -0.010 | -0.023 | -0.052 | -0.047 | -0.020 | 0.009 | 0.003 |
| Family member | -0.036 | -0.021 | -0.021 | -0.017 | -0.015 | -0.012 | -0.016 | -0.034 | -0.035 | -0.025 |
| House Ownership(ref.: rent) | | |  |  |  |  |  |  |  |  |
| Own without mortgage | -0.018 | -0.061 | -0.029 | -0.011 | -0.053 | -0.065 | -0.073 | -0.070 | -0.073 | -0.049 |
| Own with mortgage | -0.138 | -0.148 | -0.156 | -0.142 | -0.137 | -0.110 | -0.134 | -0.163 | -0.170 | -0.128 |
| Living space | 0.091 | 0.104 | 0.106 | 0.104 | 0.082 | 0.075 | 0.100 | 0.097 | 0.106 | 0.107 |
| Physical exercise | 0.033 | 0.031 | 0.032 | 0.033 | 0.042 | 0.042 | 0.033 | 0.028 | 0.031 | 0.032 |
| Relative humidity | -0.013* | -0.012. | -0.012. | -0.012. | -0.011 | -0.009 | -0.013. | -0.014* | -0.014* | -0.013* |
| Temperature | -0.004 | -0.005 | -0.006 | -0.008 | -0.007 | -0.004 | -0.002 | -0.003 | -0.005 | -0.005 |
| Population density | 0.000 | 0.000 | 0.000 | 0.000 | 0.000 | 0.000 | 0.000 | 0.000 | 0.000 | 0.000 |
| Transportation land-use density | -12.570 | 0.015 | 1.214 | 1.946. | 1.520 | 1.181 | 1.503 | 2.593* | 1.266 | 0.022 |
| Green space density | 55.160 | 0.239 | 0.559 | 1.122 | 1.233 | 1.661 | 1.694 | 1.778. | 1.203 | 0.753 |
| Open space density | -0.869 | -0.015 | -0.030 | -0.014 | 0.009 | 0.021 | 0.017 | 0.002 | -0.020 | -0.016 |
| Facilities density | 686.600 | 1278.000* | 395.000 | 592.800 | 1183.000 | 2119.000. | 1848.000 | 1177.000 | 1922.000 | 2325.000. |
| Respiratory symptoms | 0.025 | 0.022 | 0.027 | 0.031 | 0.028 | 0.024 | 0.023 | 0.025 | 0.022 | 0.022 |
| Mental disorder | 0.052** | 0.054*** | 0.051** | 0.053** | 0.052** | 0.053*** | 0.053** | 0.051** | 0.052** | 0.052** |
| R^2^ | 0.213 | 0.230 | 0.226 | 0.231 | 0.226 | 0.233 | 0.230 | 0.237 | 0.224 | 0.220 |
| Adjusted R^2^ | 0.098 | 0.117 | 0.113 | 0.118 | 0.113 | 0.121 | 0.117 | 0.125 | 0.111 | 0.106 |
| Model’s P-value | 0.011 | 0.004 | 0.005 | 0.003 | 0.005 | 0.003 | 0.004 | 0.002 | 0.005 | 0.007 |

Signif. codes: ‘*******’: 0.001; ‘******’: 0.01; ‘*****’: 0.05; ‘.’: 0.1; ‘’: 1
