# Supplementary material for: Assessment of air pollution and air quality perception mismatch using mobility-based real-time exposure
Source: PLoS One. 2024 Feb 27;19(2):e0294605. doi: 10.1371/journal.pone.0294605 (PMC10898763; doi:10.1371/journal.pone.0294605)
Supplement: Attachment — Submitted filename: Response to Reviewers_019.docx [file pone.0294605.s005.docx]

Manuscript ID: PONE-D-23-04918 [EMID:0f802a08040d887a]

**“Assessment of Air Pollution and Air Quality Perception Mismatch Using Mobility-based Real-time Exposure”**

Response to Reviewers’ comments

Dear Editor,

We appreciate your decision letter and the helpful feedback from the reviewers on our manuscript. We have carefully revised our paper to address their comments and have made corrections to address their concerns. We have submitted a version of our revised manuscript with “Tacked Changes” and an unmarked version without changes.

We are particularly grateful to you and the reviewers for your efforts in handling and reviewing our paper. The thoughtful comments provided by the reviewers have greatly assisted us in improving the paper. We look forward to hearing from you regarding our revised manuscript.

Best regards,

The Authors

--------------------------------------------------------------

**Journal Requirements:**

**[Comment 1]** Please review your reference list to ensure that it is complete and correct. If you have cited papers that have been retracted, please include the rationale for doing so in the manuscript text, or remove these references and replace them with relevant current references. Any changes to the reference list should be mentioned in the rebuttal letter that accompanies your revised manuscript. If you need to cite a retracted article, indicate the article’s retracted status in the References list and also include a citation and full reference for the retraction notice.

**(Response 1)** The authors have carefully checked the format of references and changed the first reference to the following status. We found no retracted papers in our references.

(Page 37, Line 665-667)

“1.	Yap J, Ng Y, Yeo KK, Sahlen A, Lam CSP, Lee V, et al. Particulate air pollution on cardiovascular mortality in the tropics: impact on the elderly. Environmental Health. 2019;18. doi: 10.1186/s12940-019-0476-4.”

**Reviewer(s)' Comments to Author:**

**Reviewer #4:** I did not review the initial manuscript, but see the authors have been responsive to comments. I will only give some comments to improve clarity of the paper, which is well written and done.

Specific comments:

**[Comment 1]** The number of tables is large, and some are not that informative. Table 6 could be included in tables 5. Tables 3 and 4 could be combined and shortened for the main text table and rest to a supplement. Table 2 can be summarized in the text.

**(Response 1)** Thanks for your suggestions. The authors have deleted Table 6 and combined it with Table 5 and the new table’s name is “Table 3”. We have also deleted Table 4 and combined it with Table 3, named as “Table 2” and moved some independent variables to the supplementary file, named as “S1 Table”. Additionally, we have updated the table numbers in the main text and supplementary file.

(Page 20-21, Line 365-366)

Table 3. Description of residence-based (RB) and mobility-based (MB) environmental factors and Mann-Whitney U test significance.

| Environmental exposures | | ring buffer | Mean | Std. | Min | Max | Sig. |
| --- | --- | --- | --- | --- | --- | --- | --- |
| Transportation land-use density | RB | 100m-150m | 3.10E-03 | 1.20E-03 | 2.30E-04 | 5.70E-03 | <0.001 |
|  | MB | 350m-400m | 2.70E-01 | 6.30E-02 | 7.30E-02 | 4.50E-01 |  |
| Green space density | RB | 100m-150m | 1.30E-04 | 3.80E-04 | 0.00E+00 | 2.90E-03 | <0.001 |
|  | MB | 350m-400m | 8.30E-02 | 6.70E-02 | 1.50E-03 | 3.50E-01 |  |
| Open space density | RB | 100m-150m | 6.30E-04 | 8.50E-04 | 0.00E+00 | 4.40E-03 | <0.001 |
|  | MB | 350m-400m | 1.00E-01 | 6.10E-02 | 5.60E-03 | 3.40E-01 |  |
| Facility density | RB | 100m-150m | 1.70E-04 | 1.50E-04 | 0.00E+00 | 7.60E-04 | 0.021 |
|  | MB | 350m-400m | 1.30E-04 | 7.40E-05 | 1.20E-05 | 3.10E-04 |  |
| Population density | RB | - | 1.30E+05 | 6.60E+04 | 6.00E+03 | 3.50E+05 | - |

(Page 19-20, Line 348)

Table 2. Descriptive statistics of participants’ characteristics.

| **Personal attributes** | **Code** | **Percent** | **SSP** | | **TSW** | |
| --- | --- | --- | --- | --- | --- | --- |
|  |  |  | Sample | Census | Sample | Census |
| **Gender** |  |  |  |  |  |  |
| Male | 0 | 45.2% | 43.8% | 46.7% | 46.7% | 46.3% |
| Female | 1 | 54.8% | 56.2% | 53.3% | 53.3% | 53.7% |
| **Age** |  |  |  |  |  |  |
| 18-24 | - | 18.6% | 15.2% | 14.3% | 21.9% | 15.7% |
| 25-44 | - | 48.1% | 47.6% | 42.1% | 48.6% | 38.7% |
| 45-64 | - | 33.3% | 37.1% | 43.6% | 29.5% | 45.6% |
| **Household Income (HKD)** | |  |  |  |  |  |
| < 9999 | 1 | 9.0% | 11.4% | 23.1% | 6.7% | 19.7% |
| 10000 - 19999 | 2 | 29.0% | 35.2% | 31.7% | 22.9% | 25.8% |
| 20000 - 39999 | 3 | 37.1% | 31.4% | 27.3% | 42.9% | 34.4% |
| >40000 | 4 | 24.8% | 21.9% | 17.8% | 27.6% | 20.0% |
| **Employment status** | |  |  |  |  |  |
| Employed | 0 | 75.7% | 78.1% | 74.5% | 73.3% | 78.6% |
| Student | 1 | 11.0% | 8.6% | 14.3% | 13.3% | 6.7% |
| Unemployed | 1 | 13.3% | 13.3% | 11.2% | 13.3% | 14.8% |
| **Education** |  |  |  | |  | |
| Primary and below | 1 | 2.4% | 1.9% | | 2.9% | |
| Secondary | 2 | 33.8% | 35.2% | | 32.4% | |
| Post-secondary: Diploma/ Certificate | 3 | 8.6% | 11.4% | | 5.7% | |
| Post-secondary: Sub-degree course | 4 | 3.8% | 4.8% | | 2.9% | |
| Post-secondary: Degree course | 5 | 40.0% | 36.2% | | 43.8% | |
| Master and above | 6 | 11.4% | 10.5% | | 12.4% | |
| **Marital Status** |  |  |  | |  | |
| Never Married | 0 | 52.9% | 49.5% | | 56.2% | |
| Divorced | 1 | 8.1% | 7.6% | | 8.6% | |
| Married | 1 | 36.7% | 40.0% | | 33.3% | |
| Widowed | 1 | 2.4% | 2.9% | | 1.9% | |
| Separated | 1 | 0.0% |  | |  | |

(Supplementary file)

**S1 Table. Descriptive statistics of participants’ characteristics.**

| **Personal attributes** | **Code** | **Percent** | **SSP** | **TSW** |
| --- | --- | --- | --- | --- |
| **House ownership** |  |  |  |  |
| Rent | 0 | 59.0% | 62.9% | 55.2% |
| Own without mortgage | 1 | 21.0% | 20.0% | 21.9% |
| Own with mortgage | 1 | 19.0% | 15.2% | 22.9% |
| **Living space (ft2)** |  |  |  |  |
| <100 | 1 | 3.3% | 6.7% |  |
| 100-300 | 2 | 28.1% | 37.1% | 19.0% |
| 301-500 | 3 | 44.3% | 45.7% | 42.9% |
| 501-800 | 4 | 21.4% | 9.5% | 33.3% |
| 801-1100 | 5 | 1.4% | 1.0% | 1.9% |
| >1100 | 6 | 1.4% |  | 2.9% |
| **Family member** |  |  |  |  |
| 1-2 | - | 29.5% | 42.9% | 16.2% |
| 3-4 | - | 59.0% | 50.5% | 67.6% |
| 5-6 | - | 11.4% | 6.7% | 16.2% |
| **Respiratory symptoms** |  |  |  |  |
| 0-6 | - | 46.7% | 38.1% | 55.2% |
| 7-12 | - | 48.6% | 59.0% | 38.1% |
| 13-18 | - | 2.9% | 0.0% | 5.7% |
| **Physical exercise days in one week (Physical exercise)** |  |  |  |  |
| ≤3 | - | 82.9% | 87.6% | 78.1% |
| 4-7 | - | 17.1% | 12.4% | 21.9% |

**[Comment 2]** Add justification for using linear regression with an ordinal variable (perceived air q) as dependent. It is acceptable but associated with assumptions.

**(Response 2)** Thanks for the comment. The authors added the assumption and showed the results in the main text.

(Page 15, Line 286-288)

“Since the dependent variable is normally distributed, as shown in Table 1 and verified by the Shapiro-Wilks test,”

**[Comment 3]** Some tables and figures need a bit more legend e.g. just add meaning of abbreviations MB=mobility-based, easier for the reader. In figure 1: Land use of the study area (Hong Kong)

**(Response 3)** Thanks for your suggestions. We added more words to provide clearer descriptions in the titles of figure and tables.

(Page 9, Line 184)

“Fig 1. Land use of the study area (Hong Kong)”

(Page 20, Line 365-366)

“Table 3. Description of residence-based (RB) and mobility-based (MB) environmental factors and Mann-Whitney U test significance.”

(Page 23, Line 403-404)

“Table 5. Regression results of perceived air pollution (Model 1: residence-based method (RB) and Model 2: mobility-based (MB) method).”

(Page 26, Line 449-450)

“Table 6. Logistic regression of perception mismatch with normal perception (high-high) as the reference group (Model 3: residence-based method (RB) and Model 4: mobility-based (MB) method).”

**[Comment 4]** In the abstract explain mismatch (figure 4 nicely shows that), add the dichotomization in the abstract. The choice of 15 for PM2.5 needs a bit more explanation (natural breaks??). Figure 4 illustrates it is quite reasonable.

**(Response 4)** Thanks for your comments. We added the definition of mismatch in the abstract. The authors replaced “natural breaks” with “K-means clustering” in main text to make this method more easily understood by readers.

(Page 2, Line 24-27)

“Mismatch is defined as the perception of poor air quality when the air pollution level is smaller than 15.536μg/m^3^ and the perception of good air quality when the air pollution level is larger than 15.608μg/m^3^.”

(Page 16, Line 294-295)

“Actual air quality is divided into two groups (low: 3.208μg/m³ - 15.536μg/m³, high: 15.608μg/m³-38.834μg/m³) based on using K-means clustering.”
