# Supplementary material for: Assessment of air pollution and air quality perception mismatch using mobility-based real-time exposure
Source: PLoS One. 2024 Feb 27;19(2):e0294605. doi: 10.1371/journal.pone.0294605 (PMC10898763; doi:10.1371/journal.pone.0294605)
Supplement: Attachment — Submitted filename: Response_to_reviewers025.docx [file pone.0294605.s006.docx]

**PONE-D-23-04918R2**

**Assessment of Air Pollution and Air Quality Perception Mismatch Using Mobility-based Real-time Exposure**

**PLOS ONE**

**Journal Requirements:**

Please review your reference list to ensure that it is complete and correct. If you have cited papers that have been retracted, please include the rationale for doing so in the manuscript text, or remove these references and replace them with relevant current references. Any changes to the reference list should be mentioned in the rebuttal letter that accompanies your revised manuscript. If you need to cite a retracted article, indicate the article’s retracted status in the References list and also include a citation and full reference for the retraction notice.

**(Response)** We have revised the reference list accordingly.

**Reviewers' comments:**

**Comments to the Author**

1. If the authors have adequately addressed your comments raised in a previous round of review and you feel that this manuscript is now acceptable for publication, you may indicate that here to bypass the “Comments to the Author” section, enter your conflict of interest statement in the “Confidential to Editor” section, and submit your "Accept" recommendation.

Reviewer #5: (No Response)

Reviewer #6: (No Response)

2. Is the manuscript technically sound, and do the data support the conclusions?

The manuscript must describe a technically sound piece of scientific research with data that supports the conclusions. Experiments must have been conducted rigorously, with appropriate controls, replication, and sample sizes. The conclusions must be drawn appropriately based on the data presented.

Reviewer #5: Yes

Reviewer #6: Yes

3. Has the statistical analysis been performed appropriately and rigorously?

Reviewer #5: Yes

Reviewer #6: Yes

4. Have the authors made all data underlying the findings in their manuscript fully available?

The [PLOS Data policy](http://www.plosone.org/static/policies.action#sharing) requires authors to make all data underlying the findings described in their manuscript fully available without restriction, with rare exception (please refer to the Data Availability Statement in the manuscript PDF file). The data should be provided as part of the manuscript or its supporting information, or deposited to a public repository. For example, in addition to summary statistics, the data points behind means, medians and variance measures should be available. If there are restrictions on publicly sharing data—e.g. participant privacy or use of data from a third party—those must be specified.

Reviewer #5: No

Reviewer #6: No

**(Response)** Our data cannot be shared publicly because they contain potentially identifying information that cannot be fully de-identified. Note that the dataset has participants’ GPS tracks and activity locations (including home and workplace locations), which can be used to discover the identity of individual participants using a process called spatial reverse engineering (e.g., by linking high-resolution geospatial data with other data such as census or survey data to discover the identity of specific individuals). The Survey and Behavioral Research Ethics (SBRE) Committee of the Chinese University of Hong Kong has imposed this restriction. Please contact the SBRE at [fssc02@cuhk.edu.hk](mailto:fssc02@cuhk.edu.hk) for inquiries about data availability or access.

5. Is the manuscript presented in an intelligible fashion and written in standard English?

PLOS ONE does not copyedit accepted manuscripts, so the language in submitted articles must be clear, correct, and unambiguous. Any typographical or grammatical errors should be corrected at revision, so please note any specific errors here.

Reviewer #5: Yes

Reviewer #6: Yes

6. Review Comments to the Author

Please use the space provided to explain your answers to the questions above. You may also include additional comments for the author, including concerns about dual publication, research ethics, or publication ethics. (Please upload your review as an attachment if it exceeds 20,000 characters)

**Reviewer #5: Review of PONE-D-23-04918R2 "Assessment of Air Pollution and Air Quality Perception Mismatch Using Mobility-based Real-time Exposure" by Wanying Song et al**

The authors present a cohort study on perceived air pollution and observed air pollution in Hong Kong, including different social groups. They use residential and a mobility-based geographic context approach to compare pollution exposure, several statistical models are used to analyse potential effects. The manuscript is clearly structured, well written and should be understandable for non-experts of the topic. The presented material fits in the scope of the journal and can be of interest for the readers.

I did not review the initial version, but the authors responded properly to previous reviewer's comments, and the manuscript is already in a good shape. I have a few items that require clarification (I opted for major revision as there is no moderate). I hope the authors can incorporate those into the manuscript before it can be published. I hope you will find my suggestions useful.

**[Comment 1]** Your data was achieved in summer 2021, which falls in the COVID-19 pandemic. To what extent does that influence the mobility pattern of the participants? Do weekdays reflect the normal working pattern? Is there sufficient difference in (mobile) activities between weekdays and weekends? Similar, how did air pollution in that area change compared to non-pandemic times? Were industrial activities and thus pollution reduced at the time of study?

**(Response 1)** Thank you for your suggestion. We added text on the COVID-19 pandemic and the corresponding air pollution concentration changes during our survey time in the revised manuscript to address your concerns.

(Pages 10, Lines 203-213)

“During our survey, which was conducted in low-risk periods of COVID-19 in Hong Kong, there were no significant mobility restrictions compared to previous waves. When compared to the baseline day (the median value for the five-week period of January 3 to February 6, 2020), COVID-19 community mobility reports showed a 36.5% increase in visits to grocery and pharmacy stores, a 6.4% increase in visits to residential areas, a 9.3% decrease in visits to retail and recreation venues, a 7.3% decrease in visits to transit stations, an 18.1% decrease in visits to parks, and a 5.8% decrease in visits to workplaces on average [49]. In 2020, industrial activities decreased, air pollution concentrations decreased, and air quality improved due to COVID-19 [50]. However, as the epidemic stabilized in 2021, domestic economic activities and electricity demand rebounded, resulting in similar average annual levels of PM_2.5_ (recorded by stationary monitoring stations) and increased levels of PM_2.5_ (recorded by roadside stations) compared to 2020 [50, 51].”

References:

49.	Google. COVID-10 Community Mobility Reports. 2021 [cited 2023 Sep 29]. Available at https://www.google.com/covid19/mobility/.

50.	HKSARG. Hong Kong greenhouse gas emission inventory for 2021. 2021 [cited 2023 Sep 29]. Available from https://www.info.gov.hk/gia/general/202307/04/P2023070300212.htm.

51.	HKSARG. 2013 to 2022 Annual Average Concentrations of Major Air Pollutants in Hong Kong. 2023 [cited 2023 Sep 29]. Available from: https://gia.info.gov.hk/general/202302/09/P2023020900560_412646_1_1675938001355.pdf.

**[Comment 2]** Figure 1: The two areas are hard to identify in the left side of the figure. Perhaps you could increase the line thickness of the boundaries, or place TSW and SSP close to the areas to make them easier to find.

**(Response 2)** Thanks for your suggestion. We increased the line thickness of the boundaries and attached the figure 1 separately.

**[Comment 2]** Minor question, why did you create 1415x1415m grids for the facilities, while e.g. land use was on 10x10m? 1415 seems somewhat unusual as cell size...

**(Response 2)** Thanks for your comment. The grid size was determined to reduce the processing power required when obtaining facility locations. The Nearby API can generate facilities within a 1km radius of a given location. The diagonal of a grid with a 1km width is approximately 1415 meters. Creating grids with a width of 1415 meters minimizes the spatial overlap of facilities. That is why we chose this grid size. The grid size was only used for obtaining facility information. We revised our expression to make it clearer in the revised manuscript.

(Page 13, Lines 274-276)

“We covered the spatial extent of HK with 1415m×1415m grids (note that the diagonal of a grid with a 1km width is approximately 1415 meters). We then obtained the locations of all facilities in these grids and then measured RB and MB exposures.”

**[Comment 3]** line 609: RH occurs first here, I assume you mean relative humidity? If so, perhaps just use that.

**(Response 3)** Thanks for your comment. We added the abbreviation of relative humidity “RH” at the first place this word appears.

(Page 7, Line 145)

“relative humidity (RH)”

**Reviewer #6:**

**[Comment 1]** Consider this statement from abstract: “A mismatch happens when the perceived air quality is poor but the air pollution level is lower than 15.536μg/m3 and when the perceived air quality is good but the air pollution level is higher than 15.608μg/m3.” Are the authors saying that some how pollution levels below 15.5 μg/m3 is objectively low and pollution levels above 15.6 μg/m3 are objectively high? How is this cutoff of 15.5 μg/m3 arrived at? From methods, it seems k-means clustering was used hence mentioning this as a justification for the cutoff may be warranted in the abstract.

**(Response 1)** Thanks for your comment. We mentioned using K-means clustering to classify PM2.5 value range into 2 groups.

(Page 2, Lines 26-27)

“using K-means clustering to divide PM_2.5_ value range into two groups”

**[Comment 2]** Rewrite this sentence in abstract as it is not clear what the authors mean: “People with higher exposures to MB transportation land use density and green space tend to have negative assessments of air pollution.”

**(Response 2)** Thanks for your comment. We revised this sentence.

(Page 2, Lines 35-36)

“People who are surrounded by higher MB transportation land-use density and green space density tend to perceive air quality poor”

**[Comment 3]** It is not clear what the authors are trying to say in lines 64-72. For example, in their own words, “People with lower levels of education and income were found to be likely to perceive poor air quality” and “Well-educated people were likely to be more informed about the adverse health effects of air pollution, which may lead to higher levels of perceived risk”. To me it reads like both well educated and low educated people are able to perceive low air quality levels. But perhaps authors are saying that “low educated people can perceive bad air quality but do not have sufficient information to perceive the associated risk”? Overall, I think this paragraph needs to be more crisp.

**(Response 3)** Thanks for your comments. After carefully checking, we deleted this sentence “People with lower levels of education and income were found to be likely to perceive poor air quality” to make this paragraph concise.

**[Comment 4]** Several studies looked at the issues in lines 109-112 but authors do not cite any studies. Example studies include Setton et al. 2011 and Gurram et al. 2015.

**(Response 4)** Thanks for your comment. We added the two references in our revised manuscript.

(Pages 6, Lines 108-114)

“However, using people’s residential location as the geographic context may not reveal how much air pollution they are actually exposed to because people travel to various locations and are also exposed to these non-residential contexts [25, 26]. Using only the residential location thus cannot capture how people interact with and interpret the air quality in different geographic or activity contexts [9]. People’s awareness of good or poor air quality may be influenced by environmental factors in the places they visited and at locations other than their residential neighborhoods.”

References:

9.	Bickerstaff K, Walker G. Public understandings of air pollution: the ‘localisation’ of environmental risk. Global Environmental Change. 2001;11(2):133-145. doi: https://doi.org/10.1016/S0959-3780(00)00063-7.

25.	Gurram S, Stuart AL, Pinjari AR. Impacts of travel activity and urbanicity on exposures to ambient oxides of nitrogen and on exposure disparities. Air Quality, Atmosphere & Health. 2015;8(1):97-114. doi: 10.1007/s11869-014-0275-6.

26.	Setton E, Marshall JD, Brauer M, Lundquist KR, Hystad P, Keller P, et al. The impact of daily mobility on exposure to traffic-related air pollution and health effect estimates. Journal of Exposure Science & Environmental Epidemiology. 2011;21(1):42-48. doi: 10.1038/jes.2010.14.

7. PLOS authors have the option to publish the peer review history of their article ([what does this mean?](https://journals.plos.org/plosone/s/editorial-and-peer-review-process#loc-peer-review-history)). If published, this will include your full peer review and any attached files.

If you choose “no”, your identity will remain anonymous but your review may still be made public.

Do you want your identity to be public for this peer review? For information about this choice, including consent withdrawal, please see our [Privacy Policy](https://www.plos.org/privacy-policy).

Reviewer #5: No

Reviewer #6: No

In addition to the above revisions, we shortened the digits in table 4.

(Page 20, Line 396)

**Table 4. ANOVA analysis for four factors.**

|  | Gender | Age | Income | Employment status |
| --- | --- | --- | --- | --- |
| Anova sig | 0.534 | 0.204 | 0.079 | 0.008 |
